# Periconception Maternal-Serum Vitamin D, Vaginal Bleeding, and Subchorionic Hemorrhage in Early Pregnancy

**DOI:** 10.3390/nu18142339

**Published:** 2026-07-16

**Authors:** Zeina M. Alkhalaf, Sunni L. Mumford, Enrique F. Schisterman, Robert M. Silver, Marie E. Thoma

**Affiliations:** 1 Department of Family Science, School of Public Health, University of Maryland, College Park, MD 20742, USA; 2 Department of Biostatistics, Epidemiology and Informatics, Department of Obstetrics and Gynecology, Perelman School of Medicine, University of Pennsylvania, Philadelphia, PA 19104, USA; 3 Department of Obstetrics and Gynecology, University of Utah, Salt Lake City, UT 84132, USA

**Keywords:** vaginal bleeding, subchorionic hemorrhage, vitamin D, preconception

## Abstract

**Background/Objectives**: To examine relationships between maternal-serum 25(OH)D levels at preconception and 8 weeks’ gestation and the risk of vaginal bleeding and subchorionic hemorrhage during pregnancy. **Methods**: A secondary analysis of the EAGeR Trial, which is a prospective, multisite, randomized controlled trial that enrolled 1228 women with 1–2 prior pregnancy losses. Analyses were restricted to participants who became pregnant and had 25(OH)D measured at preconception (*n* = 747) and 8 weeks’ gestation (*n* = 605). Vaginal bleeding and subchorionic hemorrhage were assessed via medical record extraction, which captured clinically recognized symptoms at medical visits measured at any point throughout pregnancy. Symptom severity of vaginal bleeding was assessed via time-varying self-reported symptoms across early pregnancy through 3–8 weeks gestation using daily diaries. Logistic regression models evaluated associations between maternal-serum 25(OH)D levels (deficient ≤ 20 ng/mL; insufficient 21–29 ng/mL; sufficient ≥ 30 ng/mL) and vaginal bleeding and subchorionic hemorrhage, while generalized estimating equations (GEE) models were used to evaluate time-varying symptom severity of vaginal bleeding from daily diary data. **Results**: Those who remained 25(OH)D deficient/insufficient had increased odds of having a subchorionic hemorrhage (aOR: 2.18; 95% CI: 1.13, 4.20) compared to those who had sufficient levels at both time points. In longitudinal GEE models, women with deficient preconception 25(OH)D had greater odds of moderate-to-heavy bleeding (vs. none) (aOR: 3.02; 95% CI: 1.13, 8.13), but no association was observed for light bleeding (aOR: 1.07; 95% CI: 0.58, 2.00). Findings were directionally similar, yet less precise when restricted to pregnancies resulting in a live birth. **Conclusions**: Persistently low vitamin D from preconception to early pregnancy is associated with increased risk of subchorionic hemorrhage and moderate/heavy vaginal bleeding, highlighting the importance of vitamin D sufficiency for implantation and early placental development.

## 1. Introduction

The periconception and early pregnancy period represent critical windows for healthy implantation and placental development [1]. Successful implantation and placentation involve complex processes that rely on optimal endometrial receptivity and disruptions to this process may result in a range of clinical outcomes, including vaginal bleeding and subchorionic hemorrhage [1]. Vaginal bleeding during the first trimester is one of the most common pregnancy complications and is experienced by 16–25% of women [2]. Sometimes bleeding during pregnancy results in subchorionic hemorrhage (SCH), when blood collects between the uterine wall and chorionic membrane, and is a frequent manifestation of vaginal bleeding between 10 and 20 weeks’ gestation occurring in approximately 11% of pregnancies [3].

One potential nutrient that has been linked to disruptions in reproductive processes is low maternal-serum vitamin D [4,5,6,7,8,9]. Low serum vitamin D may result in disruption of hormones that help to maintain the pregnancy (e.g., estrogen and progesterone), and may alter local and systemic immune functions which facilitate endometrial receptivity and implantation [6,7,8,9]. Vitamin D receptors on trophoblasts also facilitate the maintenance of pregnancy in the presence of sufficient serum vitamin D levels, possibly through anti-inflammatory effects directly in the uterus and placenta [1,6,10]. They also may operate systemically to modulate maternal immune tolerance [11,12,13,14,15,16,17].

Prior epidemiologic studies have linked low maternal-serum vitamin D to adverse reproductive outcomes, including pregnancy loss, which may also be associated with disrupted implantation and placentation [2,15,16,18,19,20]. However, few studies have examined other clinical markers of disrupted implantation, such as bleeding during pregnancy, which may occur with or without a pregnancy loss, and can be a source of considerable stress and anxiety for both women and obstetric care providers [2,3,18,21,22].

In this study, we investigate the association between maternal-serum 25(OH)D concentrations measured at preconception and 8 weeks’ gestation with risk of vaginal bleeding and subchorionic hemorrhage. We utilized prospective data from a cohort of healthy women with a history of 1–2 prior pregnancy losses, leveraging both medical records and daily symptom diaries to capture bleeding events during early pregnancy and their severity.

## 2. Materials and Methods

This is a secondary analysis of the Effects of Aspirin in Gestation and Reproduction (EAGeR) trial which enrolled 1228 healthy women in a multisite, prospective, double-blind, block-randomized, placebo-controlled clinical trial to evaluate the effect of low-dose aspirin (LDA) on live birth. Women between the ages of 18 and 40, with regular menstrual cycles and 1–2 prior pregnancy losses were eligible [23]. Women were excluded from the trial if they had any chronic use of anti-inflammatory drugs, major medical disorders (e.g., diabetes), or any prior diagnoses of infertility (e.g., polycystic ovarian syndrome, endometriosis). Women were followed for up to 6 menstrual cycles while trying to conceive, and throughout pregnancy if they conceived. Details of the study design have been published previously and the trial was registered on clinicaltrials.gov, no. NCT00467363 [24].

### 2.1. Study Design and Population

This secondary analysis included women in the EAGeR trial who became pregnant and had measured serum 25(OH)D levels at preconception (baseline) or 8 weeks’ gestation, and available data on the outcomes of vaginal bleeding or subchorionic hemorrhage throughout their pregnancy. Restriction to women who achieved pregnancy allows for capture of the outcomes of subchorionic hemorrhage or vaginal bleeding at any point in time during pregnancy. Sensitivity analyses were conducted to restrict the analysis to women who had a live birth to assess subchorionic hemorrhage and vaginal bleeding independent of any factors that may lead to a pregnancy loss. Inverse probability weights were used to account for any selection biases that could result from restriction to pregnancy or live birth using methods described previously [25,26]. Pregnancy status was determined via positive urine hCG pregnancy tests (Quidel Quickvue, Quidel Corporation, San Diego, CA, USA), conducted at home or in the clinic at the time of expected menses.

### 2.2. Vitamin D Assessment

Serum samples were collected at baseline prior to randomization to LDA and at 8 weeks’ gestation if women conceived. Samples were stored at −80 °C until analysis [23]. Combined concentrations of 25-hydroxyvitamins D2 and D3 (25(OH)D) were measured using ELISA solid-phase sandwich enzyme immunoassay (BioVendor R&D, Ashville, NC, USA), which has been validated previously [27].

The vitamin D cutoffs used in this analysis are based on levels designated by the Endocrine Society, which help to inform clinical interpretation and comparison with other studies [5]. Women were classified as 25(OH)D deficient (≤20 ng/mL [≤50 nmol/L]), insufficient (21–<30 ng/mL [52.5–<75 nmol/L]), or sufficient (≥30 ng/mL [≥75 nmol/L]) at preconception and 8 weeks’ gestation [5]. Change in serum vitamin D status from preconception to 8 weeks’ gestation was categorized as: improved (deficient/insufficient to sufficient), declined (sufficient to deficient/insufficient), deficient/insufficient: no change (remained deficient/insufficient), and sufficient: no change (remained sufficient).

### 2.3. Outcome Measures

#### 2.3.1. Vaginal Bleeding and Subchorionic Hemorrhage Medical Record Abstractions

Vaginal bleeding (yes/no) and subchorionic hemorrhage (yes/no) at any point during pregnancy were assessed through medical chart abstractions completed by study staff (Supplemental Figure S1). Due to subchorionic hemorrhage typically being diagnosed by ultrasonography in clinical care, documented subchronic hemorrhage diagnoses were presumed to reflect clinician-diagnosed ultrasound findings. However, ultrasound images and reports were not reviewed for this secondary analysis. All questionnaires were independently reviewed by two board-certified reproductive endocrinologists and a perinatal epidemiologist following the study’s completion [28]. Participants with any indication of vaginal bleeding or subchorionic hemorrhage documented in the medical record abstractions during this period were classified as having the outcome, and those with no documented indication were classified as having no bleeding.

#### 2.3.2. Vaginal-Bleeding Time-Varying Assessment of Severity Through Daily Diaries

The time-varying outcome of symptom severity of vaginal bleeding was captured through validated self-report scales via daily diaries during early pregnancy (3–8 weeks’ gestation). Diaries were completed by participants at the beginning of enrollment in the EAGeR trial (for the first two preconception cycles) and for 4 weeks once a pregnancy was achieved (gestational weeks 3–8) [29]. Daily diary questions that assessed the severity of vaginal bleeding were ““Please tell us if you had any bleeding or spotting (Supplemental Figure S2). Refer to the “Bleeding and spotting chart” to help you assess the degree of bleeding. If none, please enter “0””. Responses to the questionnaire were categorized as: 1 = none, 2 = any bleeding, 3 = spotting/very light, 4 = moderate/heavy bleeding” (Supplemental Figure S3). Vaginal bleeding severity through self-report was summarized using 2-week intervals in early pregnancy: 3–4 weeks, 5–6 weeks, and 7–8 weeks from LMP [15], and were classified in three ways: (1) any bleeding (versus none) or (2) light bleeding (versus none), or (3) moderate/heavy bleeding (versus none). Vaginal bleeding severity classifications were dichotomized for analyses, i.e., for those with light bleeding, those with moderate/heavy bleeding were excluded and for moderate/heavy bleeding those with light bleeding were excluded.

Analyses of data from medical chart abstractions and daily diaries were assessed separately and not combined in analyses.

### 2.4. Confounders

Information was collected on potential confounders, including demographic factors (e.g., age, income, race, education, employment), lifestyle characteristics (e.g., physical activity, alcohol use, multivitamin use), reproductive health factors (e.g., parity), and anthropometric measures, with BMI calculated from standardized assessments of height and weight by study staff [28]. Physical activity was assessed using the International Physical Activity Questionnaire (IPAQ) and was categorized according to the standard IPAQ scoring criteria of low, moderate, or high [30]. High physical activity was defined as vigorous-intensity activity on at least 3 days that accumulated ≥1500 MET-minutes/week, or any combination of walking, moderate-intensity, or vigorous-intensity activity on at least 7 days accumulating ≥3000 MET-minutes/week [30]. Moderate physical activity was defined as vigorous-intensity activity on at least 3 days for ≥20 min/day, or a combination of walking, moderate-intensity, or vigorous-intensity activities on at least 5 days that accumulate ≥600 MET-minutes/week [30]. Low-intensity participants were those who did not meet the criteria for high or moderate physical activity [30]. Multivitamin use was available as a self-reported variable to capture folic acid and other vitamin use; however, because dose and formulation data was not available, this variable was categorized in our analyses. No participants were classified as a “yes folic acid/no vitamins” in the analysis. Additionally, the assigned treatment (low dose aspirin/placebo), was also considered as a potential confounder given previous evidence showing an increased risk of vaginal bleeding with LDA [15,31]. Smoking in the past year was self-reported and categorized as never, less than six times per week, or daily. Alcohol consumption was self-reported in the past year and was categorized as never, sometimes, or often. Covariates were selected based on prior literature and presumed causal relationships with vitamin D status and bleeding-related pregnancy outcomes, rather than data-driven variable selection.

### 2.5. Statistical Analyses

#### 2.5.1. Descriptive Analyses

Descriptive analyses compared baseline maternal characteristics across categories of preconception 25(OH)D levels using chi-square tests for categorical variables and ANOVA for continuous variables. We then examined differences in the prevalence of vaginal bleeding and subchorionic hemorrhage reported up to 8 weeks’ gestation. Vitamin D categories were selected a priori using clinical cut points based on the Endocrine Society to support comparison with prior studies.

#### 2.5.2. Logistic Regression Chart Abstraction Models

Associations between the change in serum 25(OH)D levels from preconception to 8-week gestation and vaginal bleeding and subchorionic hemorrhage at any point during pregnancy from the medical chart abstraction were estimated using logistic regression models. Separate models were estimated for vaginal bleeding or subchorionic hemorrhage. Adjusted models controlled for sociodemographic and lifestyle covariates and include age, smoking, season, exercise, income, race, education, alcohol, parity, treatment assignment, employment, vitamin use, and BMI [25,26].

Inverse probability weights were used to account for selection bias that may occur by only including women who became pregnant (i.e., women who did not become pregnant were excluded in this analysis). Inverse probability weights models included covariates that were associated with the probability of becoming pregnant and included age, smoking, exercise, season, race, education, parity, alcohol, treatment assignment, employment, vitamin D, vitamin use, and BMI [25,26].

#### 2.5.3. Generalized Estimating Equations (GEE) Daily Diary Regression Models

We evaluated the association between changes in 25(OH)D levels and severity of vaginal bleeding during early pregnancy across 3–8 weeks of gestation using self-report data from daily diaries. We fit generalized estimating equations (GEE) with a binomial family and logit link to estimate odds ratios (ORs) and 95% confidence intervals, using an unstructured correlation matrix. These models allowed us to capture the outcome of vaginal bleeding at each biweekly interval (3–4 weeks’, 5–6 weeks’, and 7–8 weeks’ gestation), rather than assuming a single outcome across the entire period. To align timing of exposure with outcome windows, preconception 25(OH)D levels were applied to weeks 3–4, the average of preconception and 8-week levels were applied to weeks 5–6, and 8-week levels were applied to weeks 7–8. Models were adjusted for the same covariates as listed above. Separate models were estimated for each dichotomous outcome that compared symptomatic women to those who reported no symptoms and were assessed as: (1) any bleeding (vs. none), (2) light bleeding (vs. none), and (3) moderate/heavy bleeding (vs. none). Separate dichotomous GEE models were used because light bleeding and moderate/heavy bleeding may clinically represent distinct outcomes rather than points along a single severity continuum. This approach allowed us to compare each bleeding category with no bleeding, and to evaluate whether associations differed by bleeding severity. Inverse probability weights were used to account for potential selection bias of becoming pregnant and missing diary windows were treated as missing and were not imputed.

### 2.6. Sensitivity Analyses

In sensitivity analyses, we restricted models to pregnancies resulting in live birth to examine whether the associations between maternal-serum 25(OH) D and bleeding outcomes were independent of bleeding that occurs as part of a pregnancy loss. Inverse probability weights were applied to account for potential selection bias due to the additional restriction to live birth [25,26]. Inverse probability weights included covariates that were associated with achieving a live birth including age, smoking, season, physical activity, parity, treatment assignment, multivitamin use and BMI. Analyses were performed using STATA version 17.0.

## 3. Results

### 3.1. Characteristics of Participants

Among 747 pregnant participants included in the primary analysis (Table 1), 190 (25.4%) ended in pregnancy loss or a non-live birth. Among the 747 participants with measured preconception 25(OH)D levels, 377 (50%) were considered to have sufficient levels, 278 (37%) insufficient, and 92 (13%) deficient (Table 1). Compared to deficient 25(OH)D levels, women with sufficient 25(OH)D levels had the lowest mean BMI (24.5 ± 5.1), were more likely to self-report White race (98.9%), had higher educational attainment (98.9%), and had blood draws during the summer (26.5%). Maternal age was similar across 25(OH)D categories.

The prevalence of vaginal bleeding or subchorionic hemorrhage at any point during pregnancy was comparable across preconception 25(OH)D categories (deficient: 35.9%, insufficient: 35.9%, and sufficient: 34.5%) and at 8 weeks’ gestation (deficient: 40.6%, insufficient: 40.9%, and sufficient: 38.5%) (Table 2). Higher prevalence of vaginal bleeding or subchorionic hemorrhage were observed among younger women (39.5%), those who were overweight (37.1%) or had obesity (37.9%), lower household income ≤$19,999 (41.2%), and lower educational attainment (41.6%).

### 3.2. Logistic Regression Chart Abstraction Results

Women with deficient/insufficient 25(OH)D levels at both preconception and 8 weeks’ gestation had higher odds of subchorionic hemorrhage compared to women whose levels were sufficient at both time points (aOR: 2.18; 95% CI: 1.13, 4.20; Table 3). Women whose 25(OH)D levels declined from sufficient at the preconception period to deficient/insufficient at 8 weeks’ gestation had suggestive associations with vaginal bleeding (aOR: 1.59, 95% CI: 0.86, 2.92) and subchorionic hemorrhage (aOR: 1.48, 95% CI: 0.61, 3.62), compared to those who remained sufficient.

When restricting analyses to live births in sensitivity analyses, associations were attenuated, though effect estimates for the persistent deficient/insufficient remained elevated (Supplemental Table S1).

### 3.3. Generalized Estimating Equations (GEE) Daily Diary Results

In longitudinal GEE analyses, where both 25(OH)D and vaginal bleeding varied over time during early pregnancy between 3 and 8 weeks’ gestation, women with deficient 25(OH)D status had increased odds of any bleeding compared to women with sufficient 25(OH)D levels, though the estimates were imprecise (Table 4; aOR: 1.27, 95% CI: 0.74, 2.20). When bleeding severity was evaluated, deficient 25(OH)D was associated with elevated odds of moderate-to-heavy bleeding versus none (aOR: 3.02, 95% CI: 1.13, 8.13), while no association was observed for light vaginal bleeding versus none (aOR: 1.07, 95% CI: 0.58, 2.00). Insufficient 25(OH)D levels showed no meaningful associations with any bleeding severity category in women compared to sufficient 25(OH)D levels.

### 3.4. Sensitivity Analyses

In logistic regression analyses from record abstractions that were restricted to pregnancies resulting in a live birth, results were generally consistent with the main findings but less precise due to a smaller sample size. Women who remained deficient/insufficient across both preconception and 8 weeks had higher, though imprecise, odds of subchorionic hemorrhage only compared with women who remained sufficient (aOR: 1.75; 95% CI: 0.86, 3.55; Supplemental Table S1). No associations were observed for women whose vitamin D status improved or declined across time points.

In GEE analyses for daily diary symptoms during early pregnancy across 3–8 weeks’ gestation that were restricted to pregnancies resulting in a live birth, no associations were observed between vitamin D status and any bleeding or light bleeding (Supplemental Table S2). However, women with deficient levels had suggestive associations with greater odds of moderate/heavy bleeding compared to women with sufficient levels (aOR: 2.85; 95% CI: 0.81, 10.01), though confidence intervals were wide.

## 4. Discussion

In this prospective cohort study among healthy women with a prior history of 1–2 pregnancy losses, persistently low serum 25(OH)D levels from preconception through 8 weeks’ gestation were associated with an increased risk of subchorionic hemorrhage. Furthermore, women with deficient 25(OH)D levels had higher odds of experiencing moderate-to-heavy vaginal bleeding during early pregnancy. Because vaginal bleeding can also occur as part of the process of pregnancy loss, we conducted sensitivity analyses restricted to live births to distinguish bleeding events that may reflect disruptions in implantation or placentation from those that occur due to pregnancy loss. Although the estimates were less precise due to smaller numbers, associations were consistent, suggesting that low serum 25(OH)D may influence a spectrum of implantation and placentation outcomes that range from vaginal bleeding events to subchorionic hemorrhage.

Our results add to a growing body of evidence suggesting that adequate maternal 25(OH)D status may support optimal implantation [6,16,31,33]. Prior studies have identified associations between preconception 25(OH)D-deficient (≤20 ng/mL) levels and both reduced fecundability and increased risk of pregnancy loss [16,19,20,34,35]. In particular, one study by Mumford et al. using data from the EAGeR study found an association between preconception-deficient (≤20 ng/mL) maternal-serum 25(OH)D levels and risk of pregnancy loss [16]. In addition, a more recent study in a population receiving IVF treatment found that deficient (≤20 ng/mL) maternal-serum levels of 25(OH)D prior to conception lowered the rate of successful pregnancy [36].

Other studies have assessed vaginal bleeding episodes during early pregnancy and found that spotting/light bleeding tend to have different characteristics than heavy bleeding episodes [37]. This is most likely to arise from different biological mechanisms, as heavy bleeding may be related to subchorionic hemorrhage, placenta previa, abruption, or infection, in comparison to spotting/light bleeding episodes that may be connected to implantation bleeding, which is thought to occur as part of a normal process of implantation [22,37,38,39,40]. In particular, spotting/light bleeding may occur during the early pregnancy period when there is a shift in the production of progesterone to maintain the pregnancy from the corpus luteum to the fully functioning placenta [8,9,17]. Clinical distinctions in the type of bleeding and development of subchorionic hemorrhage may illuminate differences in biological mechanisms that could explain our findings and the role of early windows of exposure (preconception through early pregnancy) [21,38,41,42].

Current Endocrine Society guidance suggests following the standard Recommended Dietary Allowance of 600 IU/day, while advising against routine serum 25(OH)D testing in healthy women [43,44]. In addition, the Endocrine Society recommends empiric vitamin D supplementation during pregnancy, which includes vitamin D-fortified foods, prenatal vitamins, and/or vitamin D supplements [43,44]. The trials informing this recommendation had vitamin D doses that ranged from 600 to 5000 IU/day, with an estimated weighted average of approximately 2500 IU/day, with no routine testing recommended to test serum 25(OH)D during pregnancy [43,44].

Although our models adjusted for several demographic, lifestyle, and reproductive factors, residual confounding remains possible. Vitamin D status is closely related to dietary intake, supplement use, sun exposure, outdoor activity, skin pigmentation, ancestry, and socioeconomic factors which may not have been fully captured by the covariates added in our models. In addition, because subchorionic hemorrhage is typically clinically diagnosed via ultrasonography, symptomatic women with vaginal bleeding may have contributed to a higher outcome ascertainment. Therefore, the observed associations should be interpreted cautiously.

Future work is needed to determine whether increasing or improving maternal 25(OH)D levels prior to preconception or during early gestation is associated with the risk of subchorionic hemorrhage or vaginal bleeding. Larger prospective cohorts with repeated preconception and early gestation 25(OH)D serum measurements, along with detailed timing of bleeding and ultrasound imaging detecting subchorionic hemorrhage, may clarify whether deficient maternal 25(OH)D serum levels are related to early implantation or placentation processes.

### Strengths and Limitations

This study has several limitations worth noting. First, the study population had limited diversity, which may reduce generalizability. Another limitation in the longitudinal analysis was having to infer 25(OH)D levels for the 5–6 week gestation period as the average between preconception and 8-week gestational age, rather than a direct measure and variation in time from measurement of preconception 25(OH) D to the first 3–4 weeks of pregnancy. In addition, because the change in 25(OH)D status was based on two single serum measurements, some improvement or decline in 25(OH)D levels may reflect assay variability, within-person biological variability, regression to the mean, or measurement error, which could bias estimates for the change categories observed. Due to subchorionic hemorrhage being typically diagnosed via ultrasonography, ascertainment may have varied according to clinical imaging patterns or the level of technology used. Women with vaginal bleeding or other early adverse pregnancy symptoms may have been more likely to be evaluated via ultrasound, increasing the likelihood of having a subchorionic hemorrhage detected. If ultrasound evaluations may have differed by symptoms of the participants or 25(OH)D status, this may have contributed to differential outcome ascertainment. However, ultrasound timing and frequency were not available to assess in the analytic dataset, therefore we could not differentiate between timing, imaging patterns, and 25(OH)D status. For medical record abstraction outcomes, the exact timing of vaginal bleeding and subchorionic hemorrhage was not available, thus limiting our ability to restrict analyses involving the 8-week 25(OH) window or change in 25(OH)D status for events occurring after the 8-week serum measurement. Another limitation is the self-reporting of the severity of bleeding from the daily diaries, which may introduce measurement error or misclassification from participants. Although sample sizes were not large enough to fully distinguish bleeding associated with pregnancy loss from bleeding arising for other reasons, restricting analyses to live births showed similar magnitudes of association, although these were less precise. Additionally, we included vitamin use as a covariate in our models but could not differentiate specific doses of vitamin D from multivitamin use, diet, sun exposure, season or other factors which may have resulted in residual confounding. Furthermore, residual confounding by ancestry and skin pigmentation may also remain given that the cohort included few non-White participants, and non-White race/ethnicity was more common among women with deficient 25(OH)D than among those with sufficient 25(OH)D serum levels. Detailed smoking measures such as pack-years, cigarettes per day, or current/former smoking status were not available, and therefore there may be residual confounding by tobacco exposure that could not be excluded. Alcohol use was categorically included in the analysis; therefore, we could not evaluate dose, timing, or frequency of alcohol intake using drinks per week or grams per day. Although our models adjusted for many factors such as demographic, reproductive, and lifestyle factors, there may still be residual confounders as not all factors may have been fully captured by our variables. Furthermore, the number of subchorionic hemorrhage events was limited, and some covariate categories were sparse. Although covariates were selected based on the prior literature and DAG-informed causal assumptions rather than data-driven variable selection, adjusted estimates that are of particular interest for subchorionic hemorrhage outcome should be interpreted cautiously given the potential for overfitting, sparse-data bias, and limited precision. Finally, the study did not use gold-standard liquid chromatography-tandem mass spectrometry for vitamin D measurement; however, previous studies conducted have found vitamin D measurement results to be similar in immunoassays through the Vitamin D External Quality Assessment Scheme (DEQAS) [27,45,46]. Therefore, the ELISA solid-phase sandwich enzyme immunoassay is a precise and valid measurement for vitamin D concentrations [27].

There are many strengths of the data to highlight. Preconception and early gestation are critical time points in which an intervention may be most likely to have an effect, and few studies have data to evaluate these measures prior to and early in pregnancy. Furthermore, the longitudinal assessment is another strength as the women used daily diaries to record their symptoms prospectively and allowed differentiation of light vs. more moderate or heavy bleeding, which may be clinically different and indicate different biologic mechanisms. Importantly, the daily diaries utilized pictographs that were previously validated [29]. The use of daily diaries has been shown to provide more thorough assessment of indicators that may change frequently with time [15]. We were able to compare these findings with medical record abstractions on subchorionic hemorrhage, which is one of the leading causes of vaginal bleeding in the first half of pregnancy [3]. As such, we were able to isolate a clinical condition (that may lead to vaginal bleeding), that has been shown to be indicative of disruptions to placentation [47], thus allowing for more nuanced assessment of the potential processes by which vitamin D may affect early pregnancy complications.

## 5. Conclusions

Persistently low maternal 25(OH)D serum levels from preconception to 8 weeks’ gestation were associated with higher odds of subchorionic hemorrhage, and deficient preconception 25(OH)D serum levels were associated with moderate/heavy vaginal bleeding. These findings suggest that periconception maternal 25(OH)D status may be relevant for early placental and vascular processes, but further research is needed to confirm these associations and determine whether improving maternal vitamin D status before or during early pregnancy may influence bleeding-related complications.

## Figures and Tables

**Table 1:** Descriptive characteristics of women in the EAGeR Trial who became pregnant, stratified by preconception maternal-serum 25(OH)D status (*n* = 747).

	EAGeR-Preconception Vitamin D		
	25(OH)D Sufficient (≥30 ng/mL)	25(OH)D Insufficient (21–29 ng/mL)	25(OH)D Deficient (≤20 ng/mL)
*n*	377	278	92
Age, years			
Mean ± SD	28.7 ± 4.4	28.7 ± 4.6	28.5 ± 5.3
18–24.9	91 (24.1)	59 (21.2)	22 (23.9)
25–29.9	144 (38.2)	121 (43.5)	40 (44.5)
30–34.9	94 (24.9)	74 (26.6)	21 (22.8)
35–40.9	48 (12.7)	24 (8.6)	9 (9.8)
* BMI, kg/m^2^			
Mean ± SD	24.5 ± 5.1	26.9 ± 6.3	30.5 ± 8.6
Underweight <18.5	15 (3.9)	12 (4.3)	3 (3.3)
Normal ≥ 18.5 & <25	242 (64.2)	129 (46.4)	28 (30.4)
Overweight ≥ 25 & <30	76 (20.2)	85 (30.6)	17 (18.5)
Obese ≥ 30	44 (11.7)	52 (18.7)	44 (47.8)
Self-reported Race			
White	373 (98.9)	271 (97.8)	77 (83.7)
Non-White	4 (1.1)	6 (2.2)	15 (16.3)
Education			
≤High School	37 (9.8)	22 (7.9)	18 (19.6)
>High School	340 (90.2)	256 (92.1)	74 (80.4)
Annual Household Income			
≥$100,000	143 (37.9)	127 (45.7)	33 (35.9)
$75,000–$99,999	63 (16.7)	39 (14.0)	6 (6.5)
$40,000–$74,999	62 (16.5)	33 (11.9)	13 (14.1)
$20,000–$39,999	83 (22.0)	64 (23.0)	30 (32.6)
≤$19,999	26 (6.9)	15 (5.4)	10 (10.9)
Employed			
Yes	289 (76.7)	200 (71.9)	65 (70.7)
No	88 (23.3)	78 (28.1)	27 (29.4)
Vitamin Use			
No Folic Acid—No Vitamins	29 (7.7)	12 (4.3)	7 (7.6)
No Folic Acid—Yes Vitamins	61 (16.2)	41 (14.8)	7 (7.6)
Yes Folic Acid—Yes Vitamins	287 (76.1)	225 (80.9)	78 (85.0)
Smoking in the past year			
Never	334 (88.6)	253 (91.0)	77 (83.7)
<6 times/week	31 (8.2)	14 (5.0)	9 (9.8)
Daily	12 (3.2)	11 (3.9)	6 (6.5)
Season of blood draw			
Fall (Sep–Nov)	103 (27.3)	70 (25.2)	34 (36.9)
Winter (Dec–Feb)	76 (20.2)	64 (23.0)	25 (27.2)
Spring (Mar–May)	198 (25.9)	83 (29.9)	24 (26.1)
Summer (Jun–Aug)	100 (26.5)	61 (21.9)	9 (9.8)
* Exercise			
Low	87 (23.1)	73 (26.3)	35 (38.0)
Moderate	164 (43.5)	107 (38.5)	38 (41.3)
High	126 (33.4)	98 (35.3)	19 (20.7)
Number of previous pregnancy losses			
1	220 (58.4)	172 (61.9)	60 (65.2)
2	157 (41.6)	106 (38.1)	32 (34.8)
Alcohol consumption in the past year			
Never	231 (61.3)	207 (74.5)	68 (73.9)
Sometimes	137 (36.3)	61 (21.9)	24 (26.1)
Often	9 (2.4)	10 (3.6)	0 (0)
Treatment assignment			
Placebo	173 (45.9)	140 (50.4)	45 (48.9)
Low Dose Aspirin	204 (54.1)	138 (49.6)	47 (51.1)

Non-white participants include American Indian/Alaska Native, Asian, Native Hawaiian or Other Pacific Islander, Black or African American, more than one race, unknown or not reported * BMI—Body Mass Index. * Exercise level was measured through the International Physical Activity Questionnaire, which assessed the level of physical activity as low, moderate, or high [32].

**Table 2:** Prevalence of medical-record abstracted vaginal bleeding and subchronic hemorrhage documented among pregnant women in the EAGeR Trial *n* = 747.

Preconception 25(OH)D	*n*	Any VB or SCH *n* (%)	Vaginal Bleeding Only *n* (%)	SCH (± Bleeding) *n* (%)
Sufficient ≥ 30 ng/mL	377	130 (34.5)	112 (29.7)	18 (4.8)
Insufficient 21–29 ng/mL	278	100 (35.9)	83 (29.9)	17 (6.1)
Deficient < 20 ng/mL	92	33 (35.9)	24 (26.1)	9 (9.8)
8-week 25(OH)D				
Sufficient ≥ 30 ng/mL	341	131 (38.5)	123 (36.1)	8 (2.3)
Insufficient 21–29 ng/mL	232	95 (40.9)	93 (40.1)	2 (0.9)
Deficient < 20 ng/mL	32	13 (40.6)	8 (25.0)	5 (15.6)
Demographics				
Change in 25(OH)D from Preconception to 8 weeks				
Improved (deficient/insufficient to sufficient)	111	45 (40.5)	29 (26.1)	16 (14.4)
Declined (sufficient to deficient/insufficient)	78	33 (42.3)	24 (30.8)	9 (11.5)
No change (deficient/insufficient)	186	75 (40.3)	45 (24.2)	30 (16.1)
No change (sufficient)	230	86 (37.4)	63 (27.4)	23 (10.0)
Age, years				
18–24.9	172	68 (39.5)	60 (34.9)	8 (4.7)
25–29.9	305	106 (34.7)	96 (31.5)	10 (3.3)
30–34.9	189	61 (32.3)	50 (26.5)	11 (5.8)
35–40.9	81	28 (34.6)	23 (28.4)	5 (6.2)
* BMI, kg/m^2^				
Underweight < 18.5	30	10 (33.3)	10 (33.3)	0 (0.0)
Normal ≥ 18.5 & <25	399	134 (33.6)	120 (30.1)	14 (3.5)
Overweight ≥ 25 & <30	178	66 (37.1)	53 (29.8)	13 (7.3)
Obese ≥ 30	140	53 (37.9)	45 (32.1)	8 (5.7)
Self-reported Race				
White	722	254 (35.2)	223 (30.9)	31 (4.3)
Non-White	25	9 (36.0)	6 (24.0)	3 (12.0)
Education				
≤High School	77	32 (41.6)	28 (36.4)	4 (5.2)
>High School	670	231 (34.5)	201 (30.0)	30 (4.5)
Annual Household Income				
≥$100,000	303	110 (36.3)	95 (31.4)	15 (5.0)
$75,000–$99,999	108	37 (34.3)	35 (32.4)	2 (1.9)
$40,000–$74,999	108	33 (30.6)	32 (29.6)	1 (0.9)
$20,000–$39,999	177	62 (35.0)	52 (29.4)	10 (5.6)
≤$19,999	51	21 (41.2)	15 (29.4)	6 (11.8)
Employed				
Yes	554	191 (34.5)	164 (29.6)	27 (4.9)
No	193	72 (37.3)	62 (32.1)	10 (5.2)
Vitamin Use				
No Folic Acid—No Vitamins	48	19 (39.6)	15 (31.3)	4 (8.3)
No Folic Acid—Yes Vitamins	109	34 (31.2)	30 (27.5)	4 (3.7)
Yes Folic Acid—Yes Vitamins	590	210 (35.6)	179 (30.3)	31 (5.3)
Smoking in the past year				
Never	664	234 (35.2)	206 (31.0)	28 (4.2)
<6 times/week	54	18 (33.3)	14 (25.9)	4 (7.4)
Daily	29	11 (37.9)	8 (27.6)	3 (10.3)
Season of blood draw				
Fall (Sep–Nov)	207	87 (42.0)	78 (37.7)	9 (4.3)
Winter (Dec–Feb)	165	56 (33.9)	48 (29.1)	8 (4.8)
Spring (Mar–May)	205	66 (32.2)	56 (27.3)	10 (4.9)
Summer (Jun–Aug)	170	54 (31.7)	47 (27.6)	7 (4.1)
* Exercise				
Low	195	69 (35.4)	62 (31.8)	7 (3.6)
Moderate	309	108 (34.9)	90 (29.1)	18 (5.8)
High	243	86 (35.4)	77 (31.7)	9 (3.7)
Alcohol consumption in the past year				
Never	506	177 (34.9)	152 (30.0)	25 (4.9)
Sometimes	222	79 (35.6)	67 (30.2)	12 (5.4)
Often	19	7 (36.8)	7 (36.8)	0 (0.0)
Treatment Assignment				
Placebo	358	118 (32.9)	101 (28.2)	17 (4.7)
Low Dose Aspirin	389	145 (37.3)	128 (32.9)	17 (4.4)

8-week vitamin D missing 142 women (mostly due to early pregnancy losses). * BMI—Body Mass Index. Non-white participants include American Indian/Alaska Native, Asian, Native Hawaiian or Other Pacific Islander, Black or African American, more than one race, unknown or not reported. * Exercise level was measured through the International Physical Activity Questionnaire, which assessed the level of physical activity as low, moderate, or high.

**Table 3:** Association of change in maternal 25(OH)D status from preconception to 8 weeks’ gestation with vaginal bleeding and subchorionic hemorrhage in the EAGeR trial among women with paired 25(OH)D measurements (*n* = 605).

	Vaginal Bleeding Only			Subchorionic Hemorrhage (With or Without Bleeding)		
Change in 25(OH)D from Preconception to 8-Weeks’ Gestation	*n* = 161	Unadjusted ^1^ OR (95% CI)	Adjusted ^2^ OR (95% CI)	*n* = 78	Unadjusted ^1^ OR (95% CI)	Adjusted ^2^ OR (95% CI)
Increased: Deficient/ Insufficient to Sufficient	29 (18.0)	0.91 (0.58, 1.44)	0.89 (0.55, 1.45)	16 (20.5)	0.90 (0.47, 1.75)	0.91 (0.44, 1.89)
Decreased: Sufficient to Deficient/Insufficient	24 (14.9)	1.57 (0.88, 2.82)	1.59 (0.86, 2.92)	9 (11.5)	1.53 (0.66, 3.54)	1.48 (0.61, 3.62)
No Change: Deficient/ Insufficient	45 (28.0)	1.26 (0.79, 2.00)	1.17 (0.73, 1.88)	30 (38.5)	1.91 (1.06, 3.44)	2.18 (1.13, 4.20)
No Change: Sufficient	63 (39.1)	ref	ref	23 (29.5)	ref	ref

OR—Odds Ratio. Logistic regression models were used to assess: (1) vaginal bleeding only (no subchorionic hemorrhage) and (2) subchorionic hemorrhage (with or without vaginal bleeding) with no vaginal bleeding or subchorionic hemorrhage as the reference category. ^1^ Unadjusted for covariates and weighted to control for potential selection bias introduced by restricting to a sample of women who achieved pregnancy. ^2^ Adjusted for all sociodemographic and lifestyle covariates which included age, smoking, season, exercise, income, race, education, alcohol, parity, aspirin, employment, vitamins, and BMI and weighted to control for potential selection bias introduced by restricting to a sample of pregnancy.

**Table 4:** Maternal-serum 25(OH)D levels and vaginal bleeding episodes from 3 to 8 weeks’ gestation using Generalized Estimating Equations (GEE) regression models where 25(OH)D and bleeding were allowed to vary over time among pregnant women in the EAGeR Trial.

EAGeR Generalized Estimating Equations Regression Models for 25(OH)D and Vaginal Bleeding				
		Unadjusted-M1 ^1^ OR (95% CI)	Adjusted-M2 ^2^ OR (95% CI)	Adjusted-M3 ^3^ OR (95% CI)
Any Bleeding (vs. None)	(*n* = 1742)			
Deficient		1.14 (0.70, 1.84)	1.31 (0.78, 2.22)	1.27 (0.74, 2.20)
Insufficient		1.10 (0.81, 1.47)	1.15 (0.85, 1.60)	1.11 (0.81, 1.51)
Sufficient		ref	ref	ref
Light Bleeding (vs. None)	(*n* = 1701)			
Deficient		0.94 (0.54, 1.64)	1.10 (0.60, 2.00)	1.07 (0.58, 2.00)
Insufficient		1.16 (0.84, 1.59)	1.23 (0.89, 1.70)	1.17 (0.84, 1.63)
Sufficient		ref	ref	ref
Moderate/Heavy Bleeding (vs. None)	(*n* = 1496)			
Deficient		2.41 (1.06, 5.44)	3.00 (1.12, 7.58)	3.02 (1.13, 8.13)
Insufficient		0.77 (0.37, 1.60)	0.80 (0.39, 1.64)	0.83 (0.39, 1.80)
Sufficient		ref	ref	ref

OR—Odds Ratio, *n* corresponds to longitudinal observations (not women). Three time windows of interest (3–4 weeks gestation, 5–6 weeks, 7–8 weeks). Time-varying vitamin D: 3–4 weeks utilizes preconception 25(OH)D levels, 5–6 weeks utilizes the average between preconception and 8-week 25(OH)D, and 7–8 weeks utilizes the 8-week 25(OH)D levels. ^1^ Unadjusted model, weighted to control for potential selection bias introduced by restricting to a sample of women who achieved pregnancy. ^2^ Adjusted for all sociodemographic covariates which include age, exercise, income, race, education, parity, employment, and season and weighted to control for potential selection bias introduced by restricting to a sample of women who achieved pregnancy. ^3^ Adjusted for all sociodemographic and lifestyle covariates which included age, exercise, income, race, education, parity, employment, season, smoking, alcohol, vitamins, aspirin, and BMI and weighted to control for potential selection bias introduced by restricting to a sample of women who achieved pregnancy.

## Data Availability

The data underlying this article have been provided by the Eunice Kennedy Shriver National Institute of Child Health and Human Development (NICHD), National Institutes of Health under a data use agreement. We do not have permission to share it with other individuals, within or outside the primary author’s institution, or with commercial enterprises. Data from the EAGeR study are available to approved researchers through the data use agreement. Information about the study and data are available here: https://www.nichd.nih.gov/about/org/dir/dph/officebranch/eb/effects-aspirin (accessed on 2 May 2026). Data used in this study were created using pre-existing questionnaires, and derived variables from this data were previously returned to NICHD contacts for this project.

## Supplementary Materials

The following supporting information can be downloaded at: https://www.mdpi.com/article/10.3390/nu18142339/s1, Figure S1: Medical Chart Abstractions for vaginal bleeding yes/no and subchorionic hemorrhage under “other” as yes/no in the EAGeR Trial.; Figure S2: Instructions for Daily Diary symptom severity for vaginal bleeding in the EAGeR Trial.; Figure S3: Pictographs for Daily Diary symptom severity for vaginal bleeding in the EAGeR Trial.; Table S1: Association of change in Maternal 25(OH)D Status from Preconception to 8-weeks’ Gestation with Vaginal Bleeding and Subchorionic Hemorrhage During Early Pregnancy (3–8 Weeks)t in the EAGeR Trial among women who had a live birth in the EAGeR Trial (*n* = 557); Table S2: Maternal Serum 25(OH)D Levels and Vaginal Bleeding Episodes from 3–8 weeks gestation using Generalized Estimating Equations (GEE) Regression Models where 25(OH)D and bleeding were allowed to vary over time among Live Births: EAGeR Trial.

## Abbreviations

The following abbreviations are used in this manuscript:

GEE	Generalized Estimating Equations
25(OH)D	25-Hydroxyvitamin D
HCG	Human Chorionic Gonadotropin
EAGeR	Effects of Aspirin in Gestation and Reproduction
LBW	Low Birth Weight
DAG	Directed Acyclic Graphs
OR	Odds Ratio
aOR	Adjusted Odds Ratio
BMI	Body Mass Index
LDA	Low-Dose Aspirin
SCH	Subchorionic Hemorrhage
VB	Vaginal Bleeding
