# Supplementary material for: Periconception Maternal-Serum Vitamin D, Vaginal Bleeding, and Subchorionic Hemorrhage in Early Pregnancy
Source: Nutrients. 2026 Jul 16;18(14):2339. doi: 10.3390/nu18142339 (PMC13415651; doi:10.3390/nu18142339)

Supplemental Tables

Supplemental Table S1. Association of change in Maternal 25(OH)D Status from Preconception to 8-weeks' Gestation with Vaginal Bleeding and Subchorionic Hemorrhage During Early Pregnancy (3-8 Weeks)t in the EAGeR Trial among women who had a live birth in the EAGeR Trial (n=557)

	**Vaginal Bleeding only**			**Subchorionic Hemorrhage (with or without bleeding)**		
**Change in 25(OH)D from Preconception to 8-Week Gestation**	N=140	Unadjusted[1] OR (95% CI)	Adjusted[2] OR (95% CI)	N=72	Unadjusted[1] OR (95% CI)	Adjusted[2] OR (95% CI)
Deficient/Insufficient to Sufficient	25 (23.6)	0.86 (0.49, 1.51)	0.97 (0.52, 1.80)	14 (13.2)	1.08 (0.52, 2.27)	1.00 (0.44, 2.27)
Sufficient to Deficient/Insufficient	19 (13.2)	1.17 (0.61, 2.25)	1.20 (0.61, 2.39)	8 (11.9)	1.17 (0.48, 2.86)	1.05 (0.40, 2.71)
No Change: Deficient/Insufficient	38 (23.0)	1.01 (0.61, 1.70)	0.96 (0.57, 1.62)	28 (17.0)	1.63 (0.87, 3.05)	1.75 (0.86, 3.55)
No Change: Sufficient	58 (26.5)	ref	ref	22 (10.1)	ref	ref

*OR-Odds Ratio
Logistic regression models were used to assess: 1) vaginal bleeding only (no subchorionic hemorrhage) and 2) subchorionic hemorrhage (with or without vaginal bleeding) with no vaginal bleeding or subchorionic hemorrhage as the reference category.
[1]Unadjusted for covariates and weighted to control for potential selection bias introduced by restricting to a sample of pregnancy.
[2]Adjusted for all sociodemographic and lifestyle covariates which included age, smoking, season, exercise, income, race, education, alcohol, parity, aspirin, employment, vitamins, and BMI and weighted to control for potential selection bias introduced by restricting to a sample of pregnancy.

Supplemental Table S2. Maternal Serum 25(OH)D Levels and Vaginal Bleeding Episodes from 3-8 weeks gestation using Generalized Estimating Equations (GEE) Regression Models where 25(OH)D and bleeding were allowed to vary over time among Live Births: EAGeR Trial

EAGeR Generalized Estimating Equations Regression Models for 25(OH)D and Vaginal Bleeding				
		Unadjusted- Model 1[1] OR (95% CI)	Adjusted- Model 2[2] OR (95% CI)	Adjusted- Model 3[3] OR (95% CI)
**Any Bleeding (vs. None)**	(N=1,486)			
Deficient		0.84 (0.45, 1.55)	0.98 (0.51, 1.89)	1.10 (0.57, 2.15)
Insufficient		1.00 (0.71, 1.40)	1.04 (0.73, 1.49)	1.04 (0.72, 1.49)
Sufficient		ref	ref	ref
**Light Bleeding (vs. None)**	(N=1,467)			
Deficient		0.70 (0.35, 1.40)	0.80 (0.38, 1.67)	0.92 (0.43, 1.94)
Insufficient		1.07 (0.75, 1.53)	1.14 (0.79, 1.64)	1.13 (0.78, 1.64)
Sufficient		ref	ref	ref
**Moderate/Heavy Bleeding (vs. None)**	(N=1,285)			
Deficient		1.91 (0.60, 6.07)	2.97 (0.91, 9.70)	2.85 (0.81, 10.01)
Insufficient		0.46 (0.15, 1.41)	0.64 (0.26, 1.60)	0.76 (0.35, 1.62)
Sufficient		ref	ref	ref

[*]OR-Odds Ratio, N corresponds to longitudinal observations (not women)
3 time windows of interest (3-4 weeks gestation, 5-6 weeks, 7-8 weeks). Time Varying Vitamin D: 3-4 weeks utilizes preconception 25(OH)D levels, 5-6 weeks utilizes the average between preconception and 8 week 25(OH)D, and 7-8 weeks utilizes the 8-week 25(OH)D levels.
[1]Unadjusted model and weighted to control for potential selection bias introduced by restricting to a sample of women who had a live birth.
[2]Adjusted for all sociodemographic covariates which include age, exercise, income, race, education, parity, employment, and season and weighted to control for potential selection bias introduced by restricting to a sample of women who had a live birth.
[3]Adjusted for all sociodemographic and lifestyle covariates which included age, exercise, income, race, education, parity, employment, season, smoking, alcohol, vitamins, aspirin, and BMI and weighted to control for potential selection bias introduced by restricting to a sample of women who had a live birth.

Supplemental Figure S1. Medical Chart Abstractions for vaginal bleeding yes/no and subchorionic hemorrhage under “other” as yes/no in the EAGeR Trial.

EAGe The Effects of Aspirin in Gestation & Re	*Chart Abstraction Instruments*<br>C.2A HOSPITALIZATIONS/L&D TRIAGE/ER VISITS<br>CAC2 v 2.1 Page 2 of 4 Aug/23/2009	Study I.D

3. Presenting complaints of the mother / complaints elicited on history...................... ☐ Check if none noted

01=Yes, CHIEF complaint,   *02=Yes, elicited on history / noted in chart as present,*   *03=Not noted in chart*

a. fetal movement decreased or absent.. |__|__|

b. sent for evaluation / admission .... |__|__|

c. vaginal bleeding .... |__|__|

d. contractions.. |__|__|

e. leakage of fluid .... |__|__|

f. abdominal pain... |__|__|

g. fever / infection.. |__|__|

h. nausea / vomiting .... |__|__|

i. trauma / motor vehicle accident .... |__|__|

j. other, specify ________________________________ .... |__|__|

4. If sent for evaluation / antepartum admission indicate primary reason .... |__|__|

01=fetal monitoring   *02=size-date discrepancy*   *03=hypertension*

04=diabetes / glucose control   *05=diagnosed with fetal demise*   *06=other*

If other specify:____

5. Fetal heart tones / cardiac activity found during visit .... |__|__|

01=Present for all babies   *02=Absent for any baby*

6. Cervical dilation.. |__|__| cm

7. Objective evidence of contractions by monitor .... *01=Yes, 02=No* .... |__|__|

8. Vaginal bleeding on examination... *01=Yes, 02=No* .... |__|__|

Supplemental Figure S2. Instructions for Daily Diary symptom severity for vaginal bleeding in the EAGeR Trial.

INSTRUCTIONS

Please complete the pregnancy daily diary each day during the four weeks after your initial positive pregnancy test in the EAGeR study clinic. The Pregnancy Daily Diary contains a card for each week and a column for each day. Please answer all the questions every day, considering that each day ends at midnight. It is best to complete the card the same time every day.

***Remember,** bring your medication bottles, pill-box, samples, and the pregnancy daily diary to your next study appointment. If you cannot make an appointment, please call in advance to make other arrangements.*

Below are explanations for some questions.

DID YOU TAKE YOUR STUDY MEDICATION TODAY?

The question refers to both study treatment (Aspirin or Placebo) and the folic acid.

PREGNANCY TEST RESULTS

Did you perform a home pregnancy test today? If you did not test or cannot get a result, please enter "0". Enter "1" for positive (pregnant), or "2" for negative (not pregnant).

BLEEDING OR SPOTTING

Please tell us if you had any bleeding or spotting. Refer to the "Bleeding and Spotting Chart" to help you assess the degree of bleeding. If none, please enter "0". If you experience any blood clots please also record the details as an event in the "Illness Chart".

NAUSEA AND VOMITING

Please report any nausea or vomiting that you have experienced today. Record these symptoms regardless of the reason. If none, please enter "0".

PELVIC PAIN OR CRAMPING

Please report any nausea or vomiting that you have experienced today. Record these symptoms regardless of the cause. Please note the most severe symptom if you have more than one. If none, please enter "0".

ALCOHOL INTAKE

Please report the number of alcoholic drinks you consumed today. One drink is equivalent to one can of beer, one glass of wine, or one shot of liquor.

TOBACCO EXPOSURE

Please report any exposure to tobacco including smoking cigarettes or being passively exposed to smoking indoors (for more than 10 minutes) where other people smoked.

If you have smoked yourself and also spent time with other people smoking, enter "1". If you did not smoke but were exposed to others smoking, please enter "2".

NUMBER OF CAFFEINATED DRINKS CONSUMED

Please enter the total number of cups of caffeinated drinks you have consumed today. Caffeinated drinks include coffee (not decaf), tea, and/or caffeinated soda. Enter "0" if none. One drink is a 12 oz cup of coffee or tea or a 12 oz can of caffeinated soda. If your cup is larger than 12 oz, please estimate how many 12 oz cans it is equal to.

Supplemental Figure S3. Pictographs for Daily Diary symptom severity for vaginal bleeding in the EAGeR Trial.

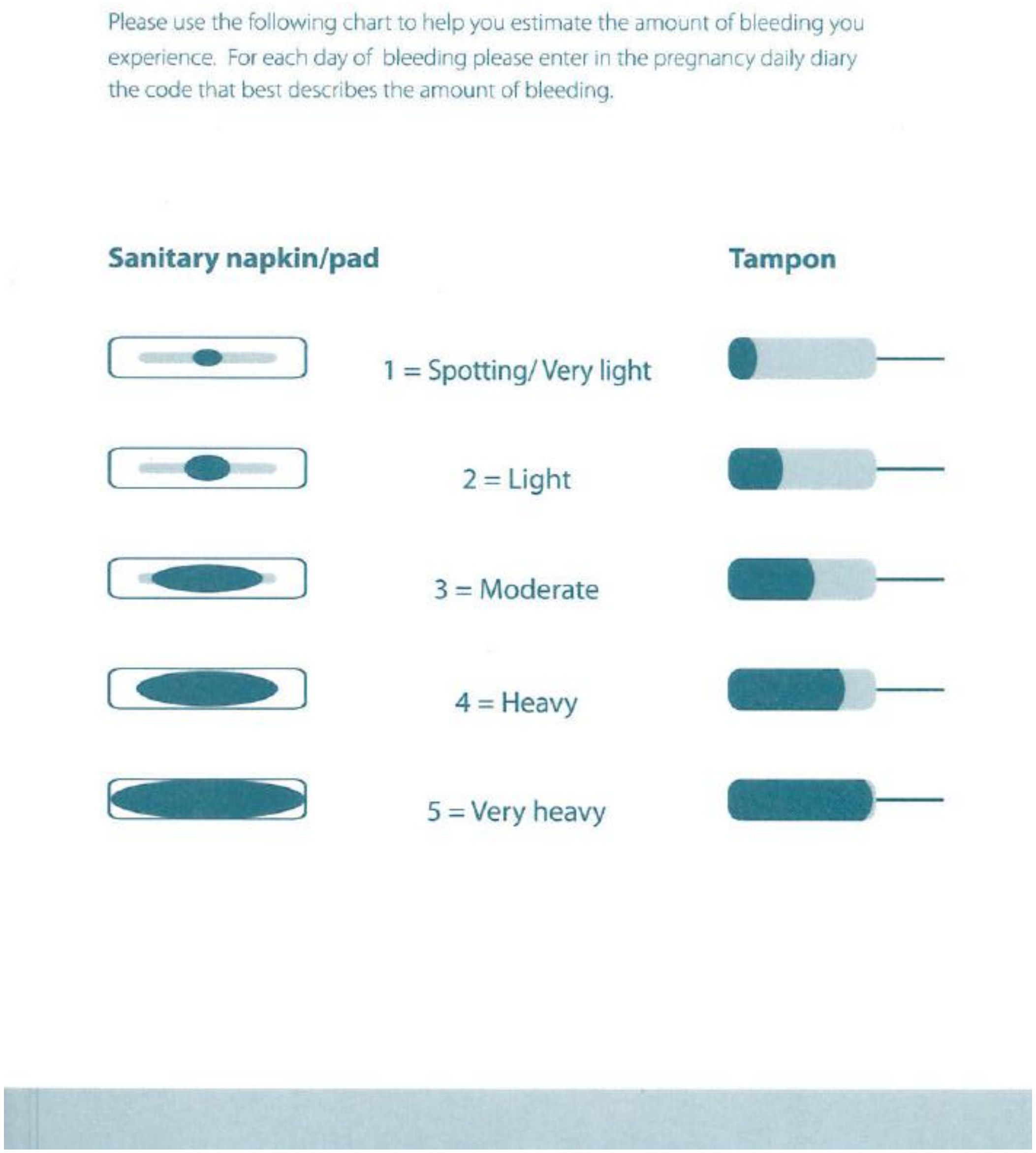

Supplement: Supplementary file 1 [file nutrients-18-02339-s001.zip › nutrients-4404711-supplementary.pdf]
